# Patient capacity for self-care in the medical record of patients with chronic conditions: a mixed-methods retrospective study

**DOI:** 10.1186/s12875-018-0852-0

**Published:** 2018-10-02

**Authors:** Kasey R Boehmer, Maria Kyriacou, Emma Behnken, Megan Branda, Victor M Montori

**Affiliations:** 1 Knowledge and Evaluation Research (KER) Unit, Mayo Clinic, 200 First Street SW, Rochester, MN 55901 USA; 2 Phoebe Family Medicine Residency, Albany, GA USA; 3 Health Sciences Research, Mayo Clinic, Rochester, MN USA

**Keywords:** Minimally disruptive medicine, Patient capacity, Electronic medical record, Electronic health record, Treatment burden, Treatment planning, Chronic conditions, Chronic illness, Multimorbidity

## Abstract

**Background:**

Patients with chronic conditions must mobilize capacity to access and use healthcare and enact self-care. In order for clinicians to create feasible treatment plans with patients, they must appreciate the limits and possibilities of patient capacity. This study seeks to characterize the amount, nature, and comprehensiveness of the information about patient capacity documented in the medical record.

**Methods:**

In this mixed-methods study, we extracted notes about 6 capacity domains from the medical records of 100 patients receiving care from 15 primary care clinicians at a single practice. Using a generalized linear model to account for repeated measures across multiple encounters, we calculated the rate of documented domains per encounter per patient adjusted for appointment type and number. Following quantitative analyses, we purposefully selected records to conduct inductive content analysis.

**Results:**

After adjusting for number of appointments and appointment type, primary care notes contained the most mentions of capacity. Physical capacity was most noted, followed by personal, emotional, social, financial, and environmental. Qualitatively, we found three documentation patterns: patients with broad capacity notes, patients with predominantly physical domain capacity notes, and patients with capacity notes mostly in domains other than physical. Records contained almost no mention of patients’ environmental or financial capacity, or of how they coped with capacity limitations. Rarely, did notes ever mention how well patients interacted with their social network or what support they provided to the patient in managing their health.

**Conclusion:**

Medical records scarcely document patient capacity. This may impair the ability of clinicians to determine how patients can handle patient work, at what point patient capacity might become overwhelmed leading to poor adherence and health outcomes, and how best to craft feasible treatment programs that patients can implement with high fidelity.

## Background

Patients with chronic conditions must mobilize their abilities and resources to access and use healthcare and enact self-care [1]. Healthcare demands, in many cases, overwhelm the capacity of patients to implement treatment programs. Furthermore, the work of healthcare competes for the same limited capacity with the work of life (e.g., from commitments to family, community, and employment) [1]. Given that capacity is limited and that people often find more meaning in these life activities than in patient work, [2] patients end up “adhering to something else.” To reduce the risk of nonadherence to healthcare, clinicians and patients must create feasible treatment plans that reflect an understanding of the role of competing demands, the overall burden of treatment, and the limited capacity patients can mobilize to routinize self-care.

To fashion personalized treatment programs and support patient self-management, clinicians need to understand the limits and possibilities of each patient’s capacity, which includes both their strengths to self-manage [3] as well as potential barriers to self-care [4]. They may have access to basic information about patient capacity in the medical record, but the extent to which information about patient capacity for self-care is available in the medical record is currently unknown. Therefore, the aim of this study was to describe the information about patient capacity extractable from the medical record, identifying its amount, nature, and comprehensiveness.

## Methods

**Fig. 1:**
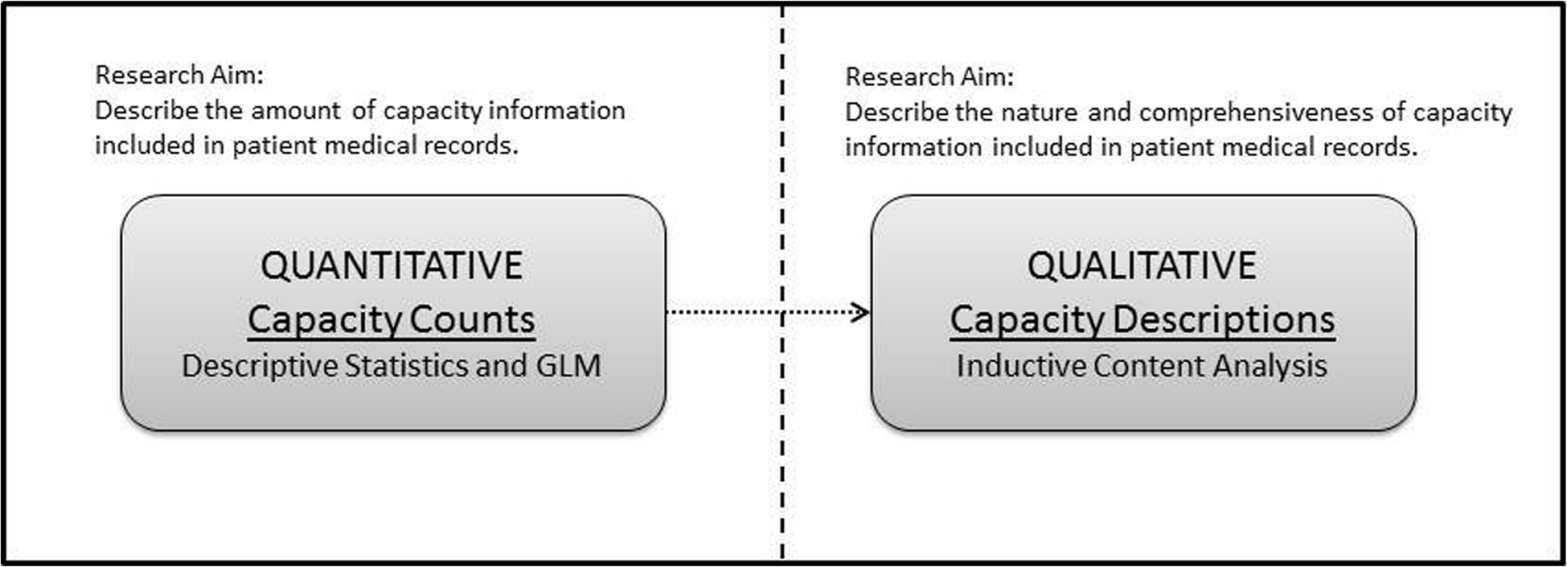
Mixed Methods Explanatory Sequential Design

This study used a mixed methods explanatory sequential design. This type of design first analyzes quantitative data and then uses those findings to inform the scope of qualitative design and analyses (Fig. 1**)** [5].

### Ethics

All study procedures were approved by the Mayo Clinic Institutional Review Board. Minnesota Research Authorization law allows patients to opt out of chart review for research. Patients who did not opt out were considered for inclusion.

### Chart selection

Eligible patients were adults 18 years or older, had at least one chronic condition, and had seen a primary care clinician in the previous six months. We defined a chronic condition as one “that lasts 12 months or more and either limits self-care or independent living or requires ongoing medical intervention.” [6] We selected six to eight charts per clinician of the most recent visits with eligible patients from the panels of all 15 primary care clinicians at a single primary care site, within a larger healthcare system, in the upper Midwest. In total, we pulled 100 charts on June 1, 2015. Because this is a sub-study of a larger project in which patients had to give written informed consent, patients who would not be able to provide such consent if approached, e.g., patients with cognitive impairment, were excluded.

### Chart review

We reviewed the chart’s latest appointment with the primary care clinician and all appointments in the previous six months, with any healthcare professional in which there was a conversation in which capacity information could be elicited. Visits included any visit in- or out-patient that occurred within the healthcare system, and included primary care, care coordination, behavioral health, and specialty care visits. Visits that were simply procedural, i.e., in-patient or out-patient surgical notes, were excluded. From each chart, we extracted appointment date(s), appointment type (ED, hospitalization, primary care, specialty, or other), number of capacity notes mentioned in each domain, and the word-for-word description of capacity information noted. To extract capacity information from the chart, we used a previously described set of categories for documenting patient capacity [7]: financial, environmental, physical, personal, emotional, and social domains. These domains specifically relate to patients’ ability to access and use healthcare and enact self-care, rather than considering more traditional clinical characteristics. For each domain, extractors had a list of possible items that could be included in each, which were determined a priori by consensus. Items documented in the clinical notes which clearly conveyed patient capacity but were not on the list, were discussed by the three extractors and added to the appropriate domain. Table 1 lists the items included in each domain.

**Table 1:** Capacity Domain Information Extracted

Physical	Pain, Fatigue, Disability, Functioning, Conditions, Symptoms, Current physical activity/exercise
Emotional	Anxiety, Depression, Grief, Worry, Stress, Coping
Social	Relationships, Family, Friends, Caregivers (paid/unpaid), Healthcare team, Volunteering, Culture, Safety in Relationships
Personal	Substance use, Smoking, Education, Self-Efficacy, Resilience, Ability to have Conversations/Make Decisions, Clinician Perceptions about Patient, Spirituality
Financial	Job, Sources of Income, Financial Commitments, Financial Difficulty, Medication Costs
Environmental	House, Neighborhood, Community, Anything about Lived Space that Affects Patient Health or Self-Care

KB, EB, and MK reviewed 10 charts in triplicate to ensure reproducible extraction, and met weekly to discuss individual extractions. We determined that after extracting the 10 charts in triplicate, good agreement was established and continued the extraction process individually until all 100 chart reviews were completed. This approach is more consistent with emergent qualitative and mixed methods designs, rather than quantitative a priori designs, which typically establish inter-rater reliability through calculation of intra-cluster correlation coefficients.

### Quantitative analysis

In addition to descriptive statistics, we estimated the number of capacity notes per domain per encounter within each appointment type (i.e. all physical capacity notes within primary care appointments was summed then divided by the number of primary care appointments). This rate was then modeled using a generalized linear model to account for repeated measures across multiple encounters. The models were adjusted by appointment type and the following domains had a link of log to account for their distribution (Financial, social, environmental and emotional). The least square means with 95% CI are reported for each capacity domain by appointment type. All quantitative analyses were conducted using SAS software (SAS Institute, version 9.4).

### Qualitative analysis

We purposefully selected the charts we would use for qualitative content analysis based upon the quantitative averages. We selected charts that had higher counts of capacity mentioned in any category than their unadjusted mean. We copied the text from each clinical note into NVivo qualitative data analysis software (QSR International Pty Ltd. Version 10, 2014). KB conducted inductive content analysis on all clinical notes [8]. This process included inductive, line-by-line coding of the entire data set, and we then synthesized these codes to summarize what was learned across all notes.

## Results

### Quantitative results

Table 2 describes the sample characteristics. The patients included in the sample had a mean age of 49 (19.9) and 52% were female. A diverse range of conditions were included in the sample. Most commonly seen conditions included type 2 diabetes, hypertension, depression, and anxiety, while less common conditions included but were not limited to irritable bowel syndrome, pain conditions, such as fibromyalgia, and asthma. The unadjusted mean of total appointments during the 6-month period was 3.4 (SD 4.0; range 1–20), with 2.1 (SD 1.7; range 1–11) of those, on average, being primary care appointments and 1.3 (SD 2.7; range 0–14) other appointment types.

**Table 2:** Sample Characteristics

Age – Mean (SD)	48.7 (19.6)
Gender - % Female	52%
Number of chronic conditions – Mean (range)	3.0 (1–10)

Table 3 describes the unadjusted mean number of times each capacity domain was documented per patient in a 6 month period. Physical capacity was by far the most mentioned domain followed by personal, emotional, social, financial, and environmental capacity.

**Table 3:** Times each capacity domain was mentioned per patient in 6 months of chart records^a^

Capacity Domain	All notes	Primary care notes only	All other notes
Physical	14.0 (21.9), 8 (4, 15), 159	9.5 (10.9), 6 (3, 12), 72	4.6 (12.5), 0 (0, 3.5), 87
Personal	5.7 (6.5), 4 (2, 6), 31	4.0 (3.6), 3 (2, 5), 25	1.8 (4.1), 0 (0, 2), 21
Emotional	3.1 (5.9), 1 (0, 4), 47	2.2 (3.0), 1 (0, 3), 17	0.9 (3.7), 0 (0, 0), 30
Social	2.6 (4.3), 2 (1, 3), 30	1.8 (1.9), 1.0 (0, 3), 12	0.8 (2.9), 0 (0,0), 18
Financial	0.9 (1.5), 1 (0, 1), 11	0.7 (0.8), 1.0 (0, 1), 4	0.3 (0.9), 0 (0,0), 7
Environmental	0.5 (1.0), 0 (0, 1), 6	0.3 (0.6), 0 (0,0), 3	0.2 (0.7), 0 (0, 0), 5

^a^Mean (Standard Deviation), Median (Inter-Quartile Range), Maximum

Table 4 shows the Least Squares Mean of capacity documentation by domain by appointment type, adjusted for number of appointments and appointment type. Primary care notes provided the most mentions of capacity across most domains, even after adjusting for number of appointments.

**Table 4:** Adjusted times capacity domain mentioned per patient, LSM (CI)*

Capacity Domain	Primary care notes	All other notes	*p* value
Physical	4.8 (4.2–5.4)	3.1 (2.4–3.7)	<.0001
Personal	2.1 (1.9–2.3)	1.3 (0.9–1.6)	0.0001
Emotional^^^	1.1 (0.9–1.4)	0.7 (0.4–1.2)	0.08
Social^^^	1.1 (0.9–1.3)	0.5 (0.3–0.9)	0.02
Financial^^^	0.41 (0.33–0.50)	0.15 (0.09–0.27)	0.001
Environmental^^^	0.22 (0.14–0.34)	0.14 (0.06–0.34)	0.37

*CI, 95% confidence interval, LSM, least square means; ^Outcome does not follow a normal distribution therefore a log link is used

### Qualitative results

**Fig. 2:**
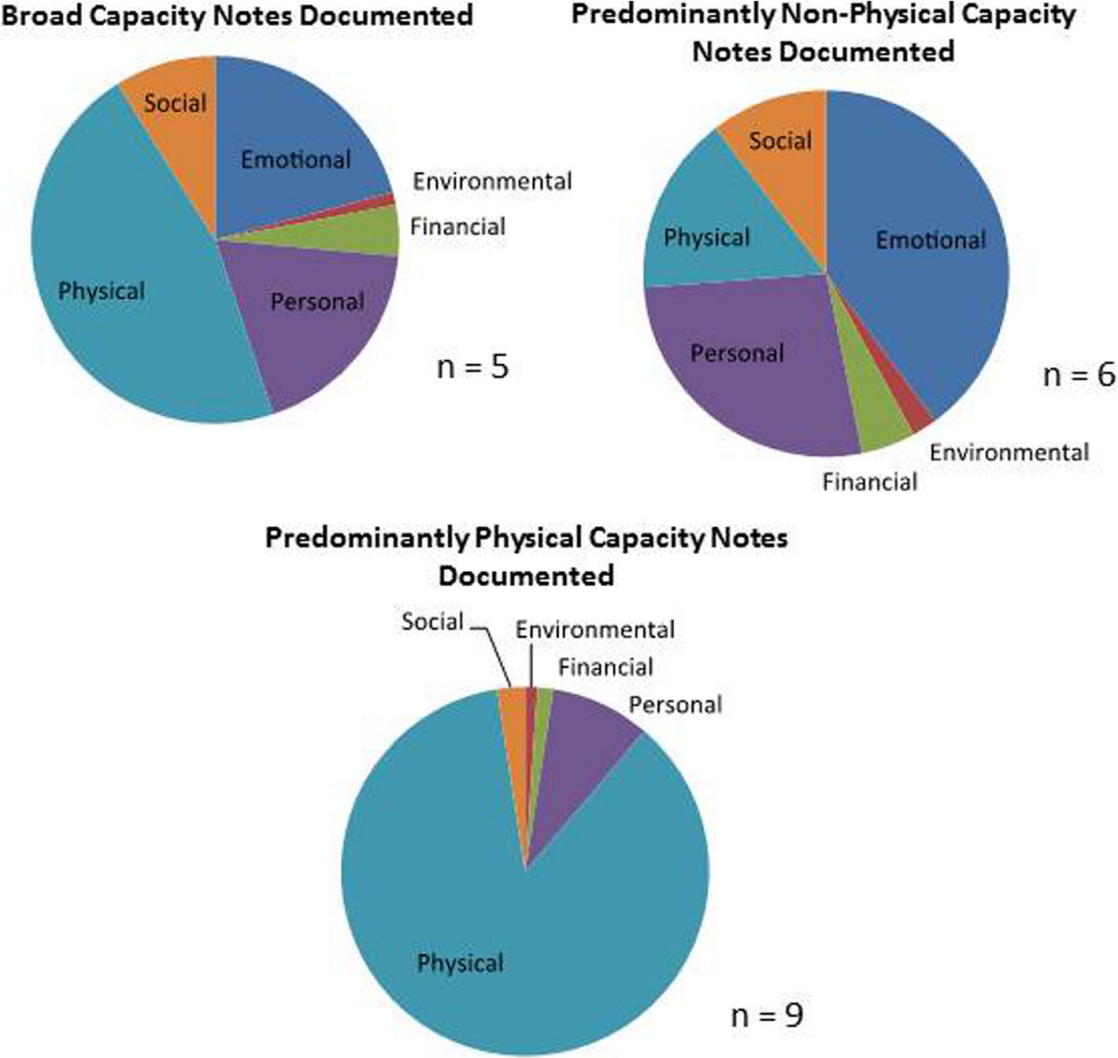
Patient Personas Represented by Capacity Documentation

Three patterns, i.e. patient “personas,” with above average capacity notes documented emerged: patients with broad capacity notes documented, patients with predominantly physical domain capacity notes documented, and patients with capacity notes documented predominantly in domains other than the physical domain (Fig. 2).

Across all patient personas, we learned little about patients’ environmental or financial capacity. For patient personas with high numbers of capacity notes documented across all domains, physical capacity notes were still most prominent, but other notes had fair representation. For patients with high numbers of capacity notes documented predominantly in the physical domain, we were able to get a good understanding of patients’ conditions, symptoms, and functional limitations, but not much about their pain and fatigue. In patients with above average capacity notes documented predominantly in domains other than physical capacity, documentation of emotional capacity problems dominated the record, particularly related to depression and anxiety, as well as occasional documentation of social situations that exacerbated these problems. Across all three personas, financial capacity was barely mentioned, mostly to note employment and the way in which patients earned or received income. Only two charts subjected to content analysis mentioned cost of care or financial difficulty. Personal capacity documentation did not refer to patients’ resilience or other personal capacity sources, but rather to patients’ ability to understand the plan of care. Social capacity had few mentions per patient, mainly to note the existence of family members or friends. Rarely, did notes ever mention how well patients interacted with their social network or what support these people provided to the patient in managing their health. Across all personas, we also had little information about patients’ abilities to cope with capacity limitations.

## Discussion

This study highlights the paucity of important capacity information documented in patient medical records. To the extent that lack of documentation reflects lack of awareness of the limits and possibilities of each patient’s capacity, medical record silence on capacity impedes clinicians’ ability to determine how patients can handle patient work, at what point patient capacity might become overwhelmed leading to poor adherence and health outcomes, and how best to craft feasible treatment programs that patients can implement with high fidelity. The medical record is particularly silent when it comes to capacity domains associated with the most disruption by illness and treatments, as uncovered in a previous survey study of patients with chronic disease: emotional capacity, physical capacity, primarily pain and fatigue, and financial capacity [2].

Given the nature of this review, it is impossible to know if the limited mention of capacity domains reflects lack of challenges in patient lives, limited assessment and discussion during clinic visits, or limitations of the documented record. This prompts the need for more in-depth conversation analysis of encounters [9] as well as testing of interventions likely to give light to these issues during consultations [10]. Given the importance of these capacity elements to patients’ ability to cope with the burden of treatment and burden of illness, careful assessment of patient capacity is necessary, with sufficient documentation to enable patient-centered team-based care.

The underwhelming documentation of capacity notes in patients with chronic conditions is particularly silent in two areas central to the work of adapting to and managing chronic disease. Both the Burden of Treatment Theory and the Theory of Patient Capacity focus on the importance of patients’ social networks in coping with illness and treatment [11, 12]. Furthermore, the Theory of Patient Capacity, as well as previous work in the experience of chronic illness, highlights the importance of patients’ biography – their personal story and the extent to which it has been disrupted by their illness [11, 13, 14]. These areas received little to no mention in the notes examined.

The findings of this study cannot be considered fully without discussing its limitations. Key study limitations include: the single-site nature of the study, convenience sampling, the extraction process, and the novelty of the extraction criteria. A single primary care clinic within a large interconnected healthcare system was chosen to undertake this study due to its inclusion as a primary site for recruitment in a subsequent prospective study to test a conversation aid intended to elicit capacity information during consultations. While this was a pragmatic choice, it limits the generalizability beyond similar primary care centers in the Midwest and does not necessarily transfer directly to health systems using other medical record systems. Patients included in this study were on clinicians’ panels for this individual site. However, we included all notes during the six-month time period regardless of location of care within the health system, and therefore, notes included hospital, specialty, and primary care received at other clinic locations as well. Second, we selected patients consecutively based upon appointment date, a convenience sample and pragmatic choice, but one which could potentially bias results given that it was not random. Next, we took steps to reduce bias as we extracted data from charts, including conducting the first set in triplicate and meeting regularly during the extraction period. While this is typical of the study design used, other more quantitatively-oriented approaches might consider the study stronger if it would have established inter-rater reliability through calculation of intra-cluster correlation coefficients. Finally, the extraction of capacity domains is novel and has not previously been undertaken. Since the time of this analysis, a more robust Theory of Patient Capacity [11] has been proposed and may warrant similar research using the theory. We have highlighted the key differences in the model used and recent theory in Table 5**.**

**Table 5:** Overlap and differences between capacity domains used and more recent Theory of Patient Capacity

Theory of Patient Capacity Constructs	Capacity Domains Model Used	Differences
Biography	Personal Emotional	The theory describes the importance of a successful reframing of the patient’s biography to include one’s condition and self-care. The theory describes that it is not specifically the existence of capacity in these domains, but how they contribute to the overall life of the patient and their ability to pursue their life’s hopes, dreams, and purpose.
Resources	Financial Physical	The theory highlights that it is not simply the presence or absence of resources, but that these resources also must be mobilized by patients. Resources captured in the theory are also more encompassing beyond not just financial or physical ones, but include those such as literacy and self-efficacy.
Environment	Environmental	The theory highlights the significant contribution of patients’ healthcare environment to their capacity, rather than considering purely home and neighborhood environment characteristics.
Work		This capacity construct is missing from the model used in this manuscript, and it highlights the contribution of experiential learning from patient work that can be accomplished, rather than patient work that is overwhelming, to patients’ capacity.
Social	Social	The theory expands upon the social capacity domain by highlighting it is not only the existence of social support in a patient’s life, but that patients are able to rely on productive, rather than detrimental, relationships in their social network for emotional and practical support in caring for their conditions.

However, despite these limitations, this study has numerous strengths, including its explanatory sequential design. This type of design is the most appropriate for studies in which the research aims seek to explain quantitative findings. Conducting the qualitative analysis after the quantitative analysis is complete allows emergent exploration, where the second phase can be designed to explore the interesting qualitative portions of the data [5]. Therefore, other mixed methods designs, such as a convergent design where qualitative and quantitative analyses are conducted simultaneously would have been weaker for this study's research aims. Furthermore, purely quantitative description of the number of times capacity was mentioned would have been less desirable.

Furthermore, this study is the first to our knowledge that explores capacity descriptions in the medical record and may prompt additional research in other settings. Much of what is captured by documenting patient capacity entails what is often named as social determinants of health. Recently, there have been calls for, expert committee guidance on, conceptual frameworks proposed, and case studies examined of how these social determinants might come to light in and be of importance to the future of medical records [15–17]. Additionally, the practice of geriatrics has been concerned with many of these capacity issue, particularly related to environment, cognition, and physical function in the measurement and documentation of frailty [18, 19]. Ultimately, what our study points to in light of these other bodies of work is that progress is needed for the benefit of patients and the care teams that support them to ensure they are able to fashion care that fits patients’ lives.

## Conclusion

This study explored the extent to which patient capacity for self-care is documented in the medical record. While mentions of patient capacity appear mostly in primary care notes, the extent is limited, mostly refers to physical capacity concerns (symptoms and functional limitations), with minimal to no mention of the state of other sources of capacity. There is significant room for improving the extent and type of capacity information documented in patient records, and the meaningful conversations and careful assessments with patients these notes should reflect, to improve patient-centered care and to implement minimally disruptive medicine [20].
